# QuickStats

**Published:** 2014-07-18

**Authors:** FROM THE NATIONAL CENTER FOR HEALTH STATISTICS

**Figure:**
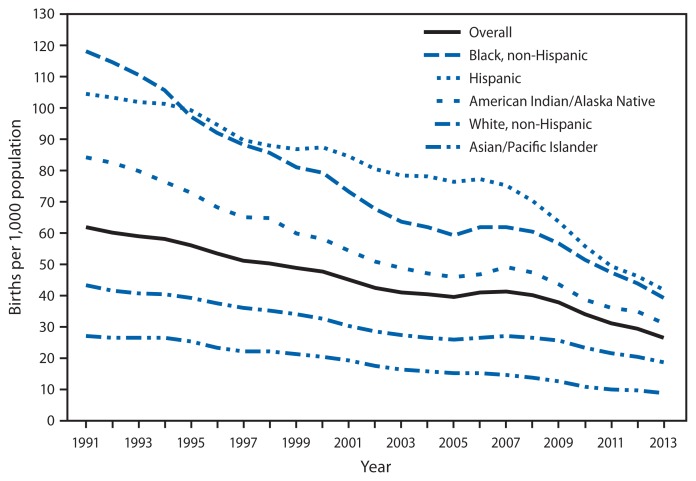
Birth Rates for Females Aged 15–19 Years, by Race/Ethnicity* — National Vital Statistics System,^†^ United States, 1991–2013^§^ * Persons categorized as American Indian/Alaska Native or Asian/Pacific Islander also might be Hispanic. Data for 1991 and 1992 for the categories non-Hispanic black, non-Hispanic white, and Hispanic exclude data from New Hampshire, which did not report Hispanic ethnicity. ^†^ Includes only U.S. residents. ^§^ Data for 2013 are preliminary.

The overall birth rate for females aged 15–19 years in the United States declined from 61.8 births per 1,000 in 1991 to 26.6 in 2013, a historic low. By racial/ethnic population, rates also declined to historic lows in 2013. Among non-Hispanic black females, the rate declined from 118.2 per 1,000 to 39.2; among Hispanic females, the rate declined from 104.6 to 41.9. Other declines were as follows: American Indians/Alaska Natives, from 84.1 to 31.2; non-Hispanic whites, from 43.4 to 18.7; and Asians/Pacific Islanders, from 27.3 to 8.8.

**Source:** Hamilton BE, Martin JA, Osterman MJK, Curtin, SC. Births: preliminary data for 2013. Natl Vital Stat Rep 2014;63(2).

**Reported by:** Brady E. Hamilton, PhD, bhamilton@cdc.gov, 301-458-4653.

